# Dr. Alvan T. Cone

**Published:** 1849-07

**Authors:** 


Died in Georgetown, Ky., on the 1st of May, 1849, Dr. Alvan T. Cone, formerly of Lee, Mass.

He graduated at the Baltimore College of Dental Surgery, in March, 1847, and removed to Georgetown, Ky., where he remained until the time of his decease.

His disease, inflammation of the brain, carried him off after an illness of three days; in his last moments, however, although in a land of strangers, being a member of the I. O. O. F., he received every attention that could be secured from the hands of that benevolent institution.

He was a young man of great personal merit, and was universally beloved and regretted by a large circle of private and professional acquaintances. R. W. A.

